# Recurrent sequence evolution after independent gene duplication

**DOI:** 10.1186/s12862-020-01660-1

**Published:** 2020-08-08

**Authors:** Samuel H. A. von der Dunk, Berend Snel

**Affiliations:** Theoretical Biology and Bioinformatics, Department of Biology, Faculty of Science, Utrecht University, Padualaan 8, Utrecht, 3584 CH The Netherlands

**Keywords:** Recurrent evolution, Independent gene duplication, Subfunctionalization, Predictability, Repeated substitutions

## Abstract

**Background:**

Convergent and parallel evolution provide unique insights into the mechanisms of natural selection. Some of the most striking convergent and parallel (collectively *recurrent*) amino acid substitutions in proteins are adaptive, but there are also many that are selectively neutral. Accordingly, genome-wide assessment has shown that recurrent sequence evolution in orthologs is chiefly explained by nearly neutral evolution. For paralogs, more frequent functional change is expected because additional copies are generally not retained if they do not acquire their own niche. Yet, it is unknown to what extent recurrent sequence differentiation is discernible after independent gene duplications in different eukaryotic taxa.

**Results:**

We develop a framework that detects patterns of recurrent sequence evolution in duplicated genes. This is used to analyze the genomes of 90 diverse eukaryotes. We find a remarkable number of families with a potentially predictable functional differentiation following gene duplication. In some protein families, more than ten independent duplications show a similar sequence-level differentiation between paralogs. Based on further analysis, the sequence divergence is found to be generally asymmetric. Moreover, about 6% of the recurrent sequence evolution between paralog pairs can be attributed to recurrent differentiation of subcellular localization. Finally, we reveal the specific recurrent patterns for the gene families Hint1/Hint2, Sco1/Sco2 and vma11/vma3.

**Conclusions:**

The presented methodology provides a means to study the biochemical underpinning of functional differentiation between paralogs. For instance, two abundantly repeated substitutions are identified between independently derived Sco1 and Sco2 paralogs. Such identified substitutions allow direct experimental testing of the biological role of these residues for the repeated functional differentiation. We also uncover a diverse set of families with recurrent sequence evolution and reveal trends in the functional and evolutionary trajectories of this hitherto understudied phenomenon.

## Background

The outcome of evolution depends on the interplay of random mutations and non-random selection. Recurrent evolutionary patterns are of general interest because they reveal chemical or physical constraints and shed light on the degree to which evolution is predictable [1, 2]. Adaptations that are genetically easily accessible can evolve many times independently. This is for instance attested by C4 metabolism in plants [3] and high-altitude adaptations in birds [4]. Traits requiring more advanced genetic rewiring are presumably less likely to evolve repeatedly, an example being echolocation in bats and cetaceans [5].

The genetic changes underlying a recurrently evolved phenotype are not necessarily recurrent themselves. In different lineages of high-altitude birds, different amino acid substitutions resulted in the same phenotypic adaptation (increased oxygen-affinity of hemoglobin [4]). Conversely, the presence of identical substitutions in different lineages does not necessarily result in recurrent evolution at the functional level, because many substitutions are functionally neutral. The latter assertion was convincingly demonstrated in two large-scale studies which found that the vast majority of recurrent substitutions in quartets of one-to-one orthologs are the result of neutral evolution and purifying selection [6, 7]. Purifying selection increases the likelihood of the same substitution happening in two distinct lineages with respect to neutrality because only a subset of mutations can be fixed.

For paralogs, the extent of recurrent sequence evolution is still unknown. This is despite the fact that gene duplication is a defining feature of eukaryotic evolution [8, 9]. Moreover, the orthology conjecture although debated [10–12] seems to suggest that paralogs are more likely to evolve different functions [13]. Therefore, recurrent sequence evolution in paralogs could potentially reflect recurrent functional change.

Functional divergence of paralogs happens in one of few ways. Often, one copy maintains the ancestral function while the other is released from selective constraints. The latter copy is then free to attain a new function (neo-functionalization), avoiding the default path towards degeneration (non-functionalization) and loss [14]. During the repeated appearance of C4 metabolism in plants such neofunctionalization took place recurrently. In at least two lineages, a non-metabolic phosphoenolpyruvate carboxylase (PEPC) independently duplicated [15] and one copy acquired specific adaptive amino acid replacements transforming it into the characteristic C4 PEPC [16]. A specific type of neofunctionalization is relocalization, whereby one of the daughter copies acquires a new subcellular localization, often through loss or gain of an N-terminal target peptide. Several protein families are known where relocalization recurred after independent duplication [17–20].

An alternative fate for duplicated genes is subfunctionalization [14, 21]. In this case, paralogs maintain the ancestral function together, conserving complementary parts of the sequence. A striking example of recurrent subfunctionalization can be found in Madbub, a mitotic checkpoint-related protein family [22, 23]. In at least ten lineages, independent duplication of the ancestral gene was followed by recurrent subfunctionalization into the Mad and Bub fates. Each of these fates conserves a distinct set of ancestral sequence motifs specifying their separate cellular roles. Thus, many diverse eukaryotes contain two copies of Madbub with this given specialization but which was independently derived. The high incidence of subfunctionalization suggests that such division of labor is adaptive in the case of Madbub. However, experiments in yeast did not detect a fitness increase, so a non-adaptive explanation should not be prematurely dismissed [24]. After a duplication, the two copies, being initially redundant, could have fixed complementary mutations such that both at some point became indispensable (the duplication-degeneration-complementation hypothesis [14, 21]). How this could have produced the pervasive recurrence in Madbub remains an enigma.

With few possible outcomes for duplicated genes, recurrent differentiation between independently duplicated paralogs could be common. Yet beside C4 PEPC, Madbub and a handful of other genes [19, 25–28], examples in the literature are scarce. Using a new framework, we automatically assay the prevalence of recurrent sequence evolution in 2883 protein families distributed over 90 diverse eukaryotes.

## Results and discussion

### Numerous cases of recurrent sequence evolution discovered by novel framework

**Fig. 1:**
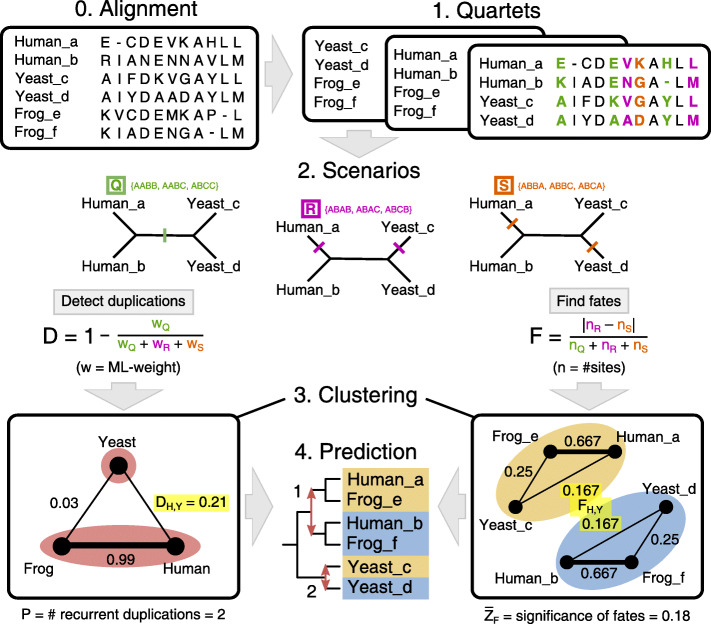
Framework for detecting recurrent sequence evolution between duplicated genes (see text or Material and Methods for details). (0) An alignment of paralog pairs serves as input. (1) The positions in the quartet support different evolutionary scenarios. (2) The two paralog pairs represent two independent duplications if scenario *Q* is most likely under a model of sequence evolution (LG +*Γ*10), yielding low *D*. Only then can we consider patterns in *R* and *S* to be recurrent substitutions (as depicted on the tree defined by *Q*). (3 & bottom left panel) Clustering based on *D*-scores of all quartets identifies independent duplications in the whole family (two in this case; denoted by red ovals). Here, human and yeast paralogs are classified as independent duplications due to a low weight for scenario *Q*, unlike human and frog paralogs. (2) For recurrent sequence evolution we require that, after the primary phylogenetic signal (*Q*), there is a clear secondary signal representing similar fates of paralogs between the two species (*F*>0). In this case, Human_a is most similar to Yeast_c and Human_b to Yeast_d (*n*_*R*_>*n*_*S*_). (3 & bottom right panel) Clustering based on *F*-scores of all quartets identifies unique fates in the whole family (two in this case; denoted by yellow and blue ovals). (4) Since yeast paralogs derive from an independent duplication event and are assigned the same two fates as human and frog paralogs, the number of duplications with recurrence (*P*) is 2. Because the shown alignment is short, the support for recurrence is low $\left (\overline {Z}_{F}=0.18\right)$

We selected gene families and subfamilies from the PANTHER database (version 9.0; [29]) where more than 10 species in our dataset possess two copies (a single paralog pair). To detect recurrent sequence evolution in these families, we developed a new framework extending on previous methods (Fig. 1; see Material and Methods for a full description). At the core are quartets consisting of two aligned paralog pairs, i.e. the paralogs from two species. Iterating through all possible quartets in a family, the duplication event is predicted from which each paralog pair originates. In the same way, the *fates* of sister paralogs—their distinctive amino acid compositions—are determined for each pair. Combining these two predictions reveals how many independent duplications were followed by similar sequence differentiation yielding the same two fates. In the case of Madbub (PTHR14030) for instance, the 28 duplicate pairs were found to be derived from 8 independent duplication events and all have obtained the same two fates. We say that Madbub shows recurrent sequence evolution with a pervasiveness of *P*=8 and a fate similarity of $\overline {Z}_{F}=6.83$ (see Fig. 1; Material and Methods).

Because we preselected families with more than 10 duplicate pairs, we retrieve at least one duplication in all of them (Suppl. Figure S1a). A subset of families has a predisposition towards retaining duplicates (Suppl. Figure S1a; see long right tail in top panel). By far most duplications are taxon-specific (Suppl. Figure S1b). Older duplications where all species still have two copies are scarce, because copies can be lost or they can go through additional rounds of duplication; in both cases, the genes are ignored in the current framework.

**Fig. 2:**
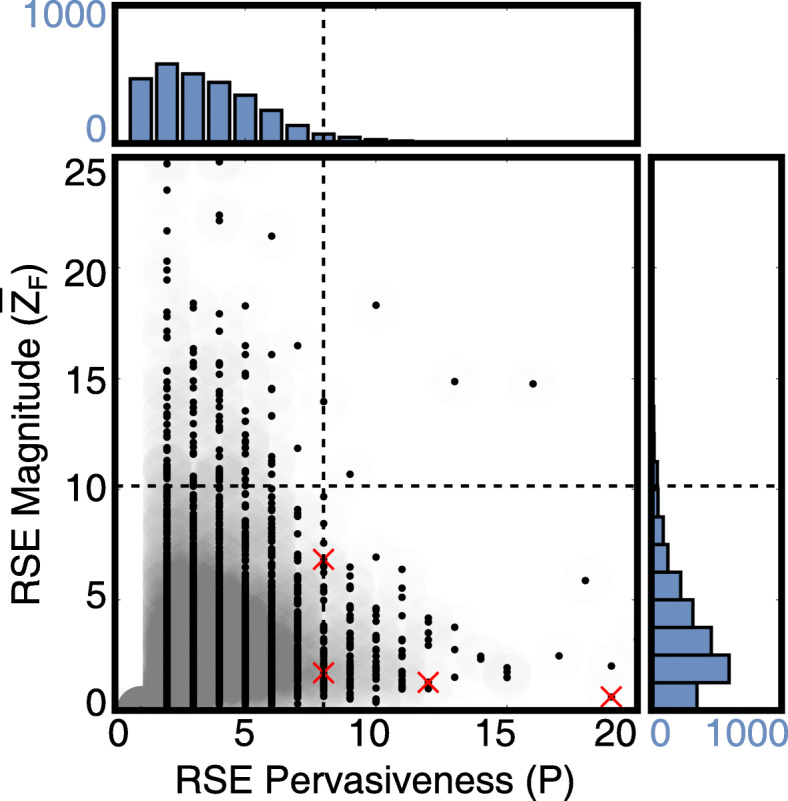
Many new cases are detected of prevalent recurrent sequence evolution (RSE) between paralogs in eukarotes. Pervasiveness and magnitude of recurrent sequence evolution are plotted for each family, with averages $\langle P\rangle =3.7, \langle \overline {Z}_{F}\rangle =3.4$ for *N*=2883. Distributions are shown in the right and top panel. Dotted lines denote top 5% of distributions. Red crosses indicate the following families: (from left to right) Hint1/Hint2 (bottom), Madbub (top), Sco1/Sco2 and vma11/vma3

Recurrent evolution left a mark on numerous eukaryotic proteins (Fig. 2; Table 1; full list in Suppl. Data). In many families (2387 of 2883, or 82.8%), at least two independent duplications occurred that were followed by similar sequence differentiation of paralogs. A considerable number of families experienced recurrent sequence evolution more pervasive than Madbub. In some cases, paralogs from many different duplications (up to 20) show recurrence at the sequence level. Nonetheless, Madbub displays relatively high fate similarity between those independent duplicates that are represented in the data $\left (\overline {Z}_{F}=6.83\right)$. This is because subfunctionalization of Madbub involves the complementary loss or divergence of substantial pieces of the sequence, such as the entire kinase domain (conserved in Bub1-like paralogs) and the KEN box (conserved in Mad3-like paralogs; see also Suppl. Figure S2; [23]).

**Table 1:** Families with the most pervasive recurrent sequence evolution (*P*≥12)

**P**	**Fate ‘yellow’**	**Fate ‘blue’**	**Common denominator**	**Potential differentiation**
20	eef-1B.1 (*Cele*)	eef-1B.2 (*Cele*)	elongation factor	-
19	cka1 (*Scer*)	cka2 (*Scer*)	casein kinase II, catalytic subunit	i.a. Specific protein interactions [36]
19	vma11 (*Scer*)	vma3 (*Scer*)	V-ATPase subunit	Complementary loss of interaction interfaces [34]
18	ercc3 (*Gthe*)	unknown	TFIIH subunit XPB	-
17	Hspa2	Hspa8	heat-shock protein	Constitutive vs. heat-induced expression [30]
16	RpIIIc160 (*Dmel*)	unknown	DNA-directed RNA polymerase subunit	-
15	Rrm2	Rrm2b	ribonucleotide reductase regulatory subunit	Complexes specialized to cell-cycle progression vs. DNA repair [35]
15	Sphk1	Sphk2	sphingosine kinase	Antagonists in sphingolipid metabolism? [37].
15	eno1 (*Xtro*)	eno4 (*Xtro*)	enolase	-
14	aspS1 (*Ddis*)	aspS2 (*Ddis*)	aspartate–tRNA ligase	-
14	unknown	unknown	ethanolaminophosphotransferase	-
13	Senp6	Senp7	sentrin-specific protease	Some substrate specificity and differences in catalytic activity [38]
13	Mpdu1	Pqlc3	∼ cargo receptors	-
13	unknown	unknown	isoleucine–tRNA ligase	-
13	Hars	Hars2	histidine–tRNA ligase	Cytoplasmic vs. mitochondrial specialization [32]
12	rpa1 (*Gthe*)	rpa1 (*Gthe*)	replication protein A1	-
12	unknown	unknown	cystinosin	-
12	nmd3 (*Gthe*)	nmd3 (*Gthe*)	60S ribosomal export protein NMD3	-
12	Sco1	Sco2	synthesis of cytochrome c oxidase	-
12	cycs (*Xtro*)	cyct (*Xtro*)	cytochrome c	Somatic vs. testis-specific in animals [39]
12	aimp1 (*Drer*)	aimp1 (*Drer*)	ARS-interacting multifunctional protein^a^	-
12	psenA (*Ddis*)	psenB (*Ddis*)	presinilin	-

^a^ARS: aminoacyl-tRNA synthetase complex

For each family we display *P*, two representative genes from each fate, their united functional annotation and their potential functional differentiation based on the literature. Human names start with a capital; other organisms are given in brackets (*Cele*: *Caenorhabditis elegans*, *Scer*: *Saccharomyces cerevisiae*, *Xtro*: *Xenopus tropicalis*, *Ddis*: *Dictyostelium discoideum*, *Drer*: *Danio rerio*, *Dmel*: *Drosophila melanogaster*, *Gthe*: *Guillardia theta*)

While families with large fate similarity $\left (\overline {Z}_{F}\right)$ may represent interesting cases of highly significant recurrent sequence evolution, we will focus here on families with high pervasiveness (*P*). Recurrent evolution in coding regions reflects recurrence at the functional level in a variety of ways (Table 1). For instance, two human paralogs Hspa2 and Hspa8 encode heat-shock proteins that differ in their expression; one is heat-inducible and one is constitutively expressed [30, 31]. Two other human paralogs, Hars and Hars2, code for copies of a histidine–tRNA ligase that differ in their subcellular localization; one is localized to the cytoplasm and one to the mitochondrion [32, 33]. Other duplicate pairs code for two members of the same complex (vma11 and vma3 in yeast [34]; see “Possible Recurrent Complexification of a Molecular Machine”) or for the same member of two homologous complexes (Rrm2 and Rrm2b in human [35]). In those cases where protein functions are known, sister paralogs have obtained different functional roles. Our framework thus uncovered recurring genotypic variation that is potentially biologically relevant.

### Duplicated genes tend to diverge asymmetrically

**Fig. 3:**
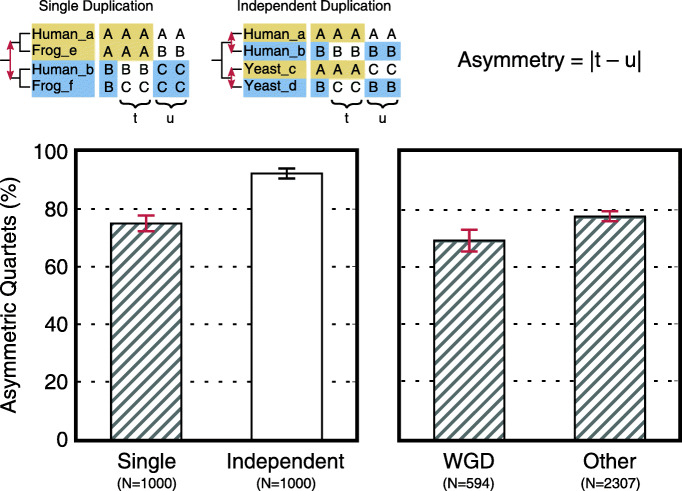
Asymmetric divergence of duplicated genes. Two primary categories of quartets are sampled: those with a single duplication and those with independent duplications (top). As an example, two quartets from Fig. 1 are shown. Within the subset of positions that supports the predicted fates (*R* in the case of human and yeast), asymmetry captures the difference in congruity between the two fates, i.e. are only Human_a and Yeast_c similar at a number of positions (*t*=ABAC) or are Human_b and Yeast_d similar as well (*u*=ABCB)? From the asymmetry (*A*), its significance *Z*_*A*_ can be calculated in the same manner as *Z*_*F*_ (see Material and Methods and Suppl. Figure S3). The bottom panels show the percentage of quartets with |*Z*_*A*_|>1.96 (i.e. significant asymmetry) as a fraction of quartets with significant fate similarity (|*Z*_*F*_|>1.96). Bars display averages; error bars give standard errors of the proportions; sample sizes are indicated beneath the categories. Note that duplications represented in Other quartets are likely substantially older than those in WGD quartets which only contain the vertebrate 2R and 3R WGD and the yeast WGD (∼500, 350 and 150 Mya)

Next, we wanted to learn more about the patterns of recurrent sequence evolution. In particular, we asked whether the two main fates in a family were symmetric or asymmetric: Do both sister paralogs carry unique pieces of sequence with high similarity between species? Intuitively, the differentiation in Madbub appears symmetric because Bub1-like copies share a conserved kinase domain and Mad3-like copies share a conserved KEN box and several other motifs. Asymmetry was measured in sampled quartets of paralogs by the number of recurrent substitutions that are asymmetric with respect to one of the two fates (Fig. 3; Material and Methods). Having already predicted the tree topology for all quartets in our families, we can distinguish those that comprise one ancestral duplication and those that comprise two independent duplications. The former reflects the trajectory of paralogs in general (analogous to methods in [40–42]). For a small subset of species, these Single Duplication quartets can be further divided into quartets derived from a whole genome duplication and those not derived from a whole genome duplication (WGD and Other, respectively; see Material and Methods). This can inform on potential confounding effects in the analysis stemming from the lumping of duplications that were created by different mutational mechanisms. Specifically, ohnologs (paralogs derived from whole genome duplications) are hypothesized to undergo qualitatively distinct functional divergence [43–46].

Most eukaryotic duplications (74.8±2.7*%*, mean ± standard error; Fig. 3) have resulted in two paralogs with a different level of conservation in their subsequent phylogeny. Fates containing independently duplicated paralogs tend to be asymmetric even more often (92.0±1.7*%*; Fig. 3). These numbers are in conflict with several previous studies that measured the evolutionary rate asymmetry between recently duplicated genes [47–49] though not with all [50]. Duplicates that diverged more recently have fewer positions that can potentially be counted as a recurrent substitution. Between single duplication quartets from WGD and those not from WGD, an age difference indeed explains most of the inequality in asymmetric tendency (Suppl. Figure S4). However, between single and recurrent duplication quartets, the latter’s greater tendency towards asymmetry is not attributable only to different sample sizes (i.e. numbers of divergent positions; Suppl. Figure S3).

The prevalence of asymmetry could reflect a high rate of neofunctionalization with respect to subfunctionalization (cf. [51]). For many genes, subdividing function is impossible; metabolic enzymes that lose their reaction center also lose their enzymatic function. Even when subdivision is possible, functions do not appear as equal sequence chunks. In Madbub, the kinase domain dominates the signal for recurrent sequence evolution, so most quartets in that family are classified as asymmetric. As a consequence of the complex relation between genotype and phenotype, asymmetric sequence divergence does not guarantee neofunctionalization. Similarly, symmetric sequence divergence does not guarantee subfunctionalization. Rather, there is a continuum: from highly asymmetric cases strongly suggesting neofunctionalization to highly symmetric cases strongly suggesting subfunctionalization and with many unresolved cases in between (Suppl. Figure S3).

### Subcellular relocalization frequently explains recurrent sequence evolution

**Fig. 4:**
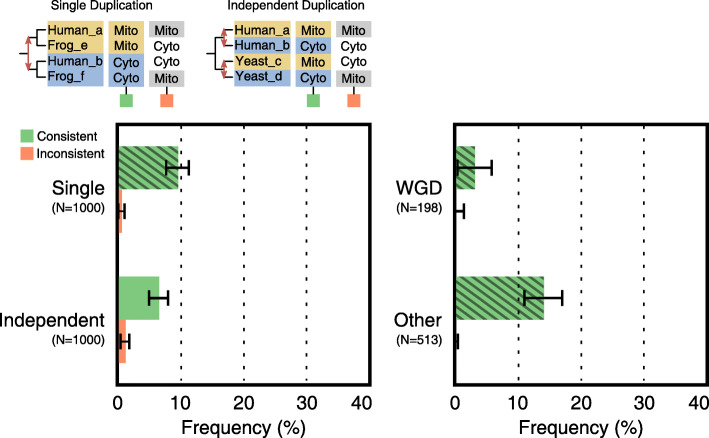
Subcellular relocalization explains many cases of recurrent sequence evolution. Quartets of specific categories were sampled from all families (see Fig. 3). Besides adhering to one of these categories, quartets are selected if there is significant fate similarity (|*Z*_*F*_|>1.96) and if there is a reliable prediction of subcellular localization for all four genes (TargetP Reliability Class <3). (Bottom) Fraction of quartets with consistent (green) and inconsistent (orange) subcellular localization patterns (configurations shown at the top). Other patterns are not shown for clarity; 70.8% of Independent Duplication quartets had a uniform localization and 16.7% contained only one gene with a distinct localization. In these cases, the functional change driving recurrent sequence evolution remains unknown. Bars display averages; error bars give standard errors of the proportions; sample sizes are indicated beneath the categories

For some duplicated genes, as mentioned previously, functional differentiation entails the acquisition of different subcellular localizations [18–20]. This may apply in particular to families that duplicated early in eukaryotic diversification, as the Last Eukaryotic Common Ancestor (LECA) had not yet finished compartmentalizing its full proteome [18]. To find cases of recurrent relocalization in our data, we predicted the subcellular localization of the four genes in sampled quartets using TargetP [52]. The two most relevant types of configurations for subcellular localization are the ones that are consistent with the predicted fates and the ones that are inconsistent with them (Fig. 4). For all quartets—irrespective of the pairs originating from one or two duplication events—consistent configurations are an order of magnitude more frequent than inconsistent configurations, corroborating our fate prediction.

Recurrent relocalization was observed in 6.4±1.5*%* (mean ± standard error) of quartets comprising two independent duplications with significant fate similarity (Fig. 4). For paralogs arising from the same duplication, a persistent relocalization was found in 9.4±1.8*%* of quartets. Hence, for a small but meaningful fraction of quartets displaying recurrent sequence evolution a functional differentiation can be inferred. Considering that TargetP only recognizes four different subcellular localizations, including plastids which many eukaryotes do not posses, more such fates could be present. The difference between single duplication quartets containing ohnologs and those with non-ohnologs again is in line with the fact that the latter are older. As with asymmetry, older duplicates have had more time to gain differential subcellular localization.

The current framework successfully identified previously described cases of recurrent relocalization: Hint1/Hint2, Ppa1/Ppa2, Shmt1/Shmt2 and Idh1/Idh2 (see Suppl. Data). For Hint, Shmt and Idh, Szklarczyk & Huynen (2009) had previously found that two independent duplications took place in the ancestors of yeast and human, followed by recurrent differentiation of the paralogs into a cytoplasmic and a mitochondrial copy [19]. For Ppa, Espiau et al. (2006) showed that three independent duplications created the paralogs in yeast, human and the excavate parasite *Leishmania major*, all of which obtained differential subcellular localization [17]. Not only did our methodology retrieve these known duplications, we also identify many additional duplications in different lineages with the same outcome (5 in Ppa, 6 in Hint, 7 in Idh and 9 in Shmt). This striking observation suggests that detection of independent evolution in two lineages is predictive for recurrent functional evolution in many more lineages. Below we illustrate one example, Hint1/Hint2, in more detail.

### Diverse modes of recurrent sequence evolution

**Fig. 5:**
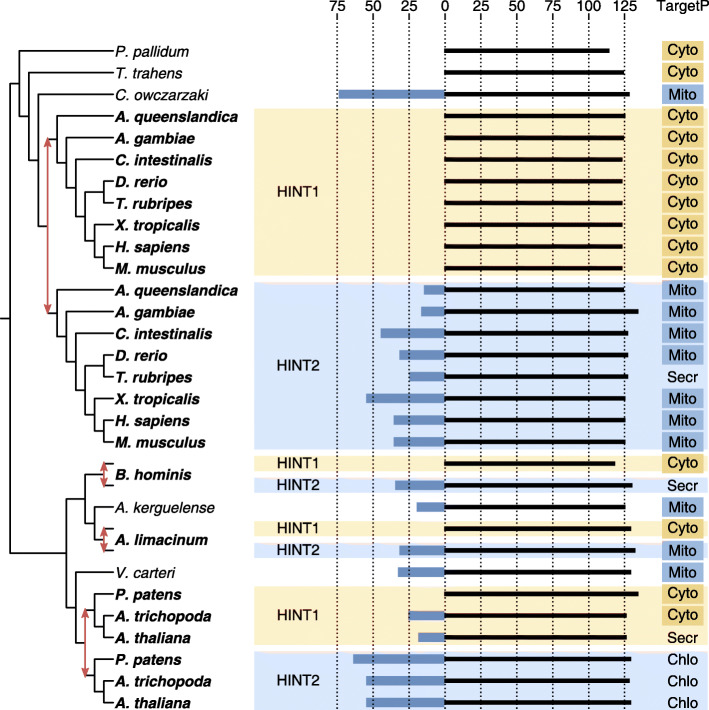
Reconciled gene tree of Hint1/Hint2. As mentioned in the main text, only unambiguous parts of the tree are shown. Red arrows indicate duplication events; species names of duplicates are shown in bold. Next to the tree, a schematic view of the recurrent pattern is shown. Shaded areas indicate the predicted fate (Hint1 and Hint2 after the human genes). The sequences are shown as horizontal bars, aligned at position 1 (Met) of human Hint1. Residues that appear N-terminal to this are represented by a thick blue bar. On the right, the subcellular localization as predicted by TargetP is shown (Mito: mitochondrion, Chlo: chloroplast, Secr: secretory, Cyto: cytoplasm)

**Fig. 6:**
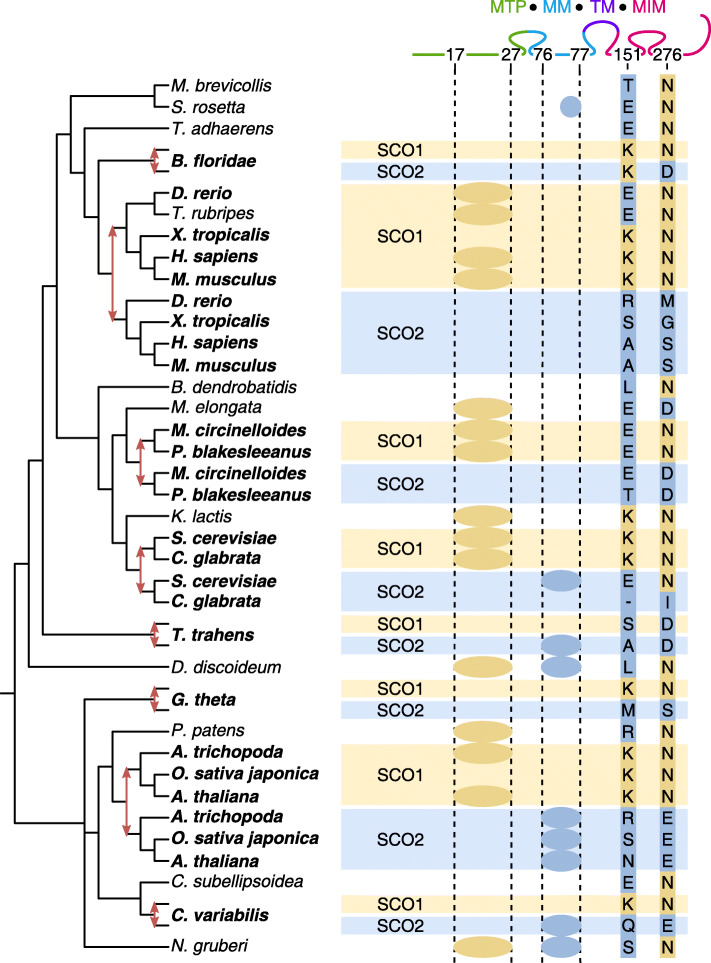
Reconciled gene tree of Sco1/Sco2 (see Suppl. Figure S5 for the full gene tree). There are four elements in the alignment that give high support for the Sco1–Sco2 differentiation: two small indels in the mitochondrial target peptide (MTP) and mitochondrial matrix region (MM), and two substitutions in the mitochondrial intermembrane section (MIM) which corresponds to the thioredoxin-like fold. Their colors show with which fate they are associated. For the two substitutions, a specific residue is associated with Sco1 (lysine at position 151 and asparagine at position 276, with human Sco1 as reference), whereas Sco2 is not associated with a specific residue. At the top, the different protein regions are shown (TM: trans-membrane)

**Fig. 7:**
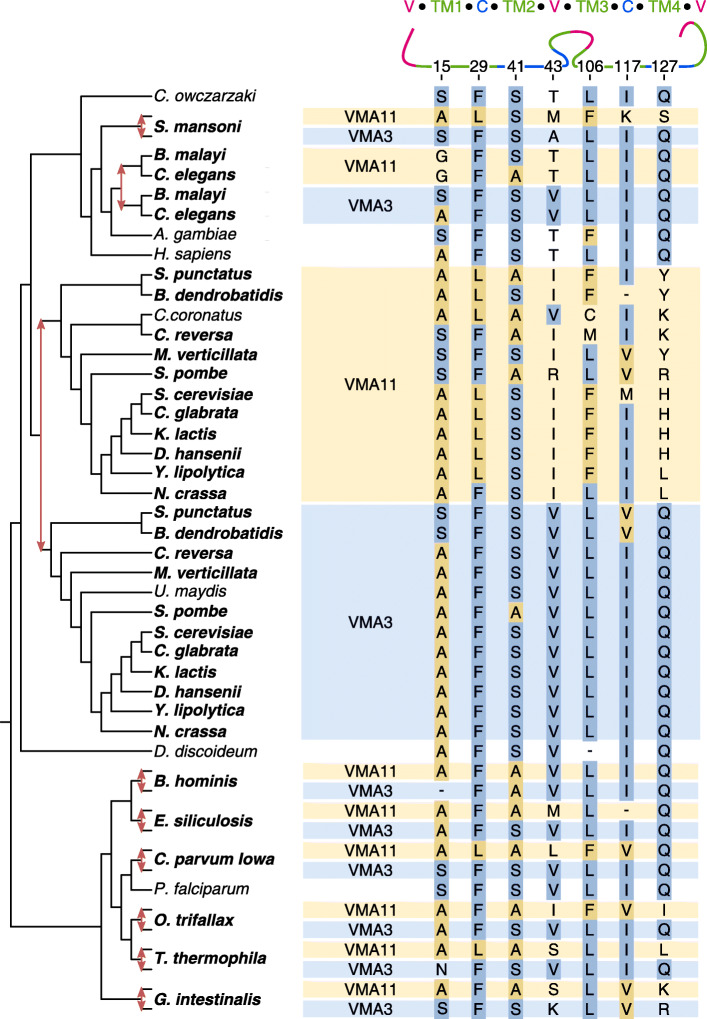
Reconciled gene tree of vma11/vma3 (V-ATPase subunits c’ and c). Seven positions with the most consistent differentiation between vma11 and vma3 are shown; the most common residue in each fate is colored according to that fate. At the top, position numbers are shown (using yeast vma11 as a reference) and the different protein sections are displayed schematically (V: vacuolar, TM: trans-membrane, C: cytoplasmic). The two cytoplasmic sections are thought to provide the interaction interfaces with adjacent subunits. See Suppl. Figure S6 for the full gene tree

The framework we have presented allows us to go back to the alignment and identify the residues that underlie the fate prediction (see Material and Methods; Suppl. Figure S2). This can serve as a validation for the *P* and $\overline {Z}_{F}$ measures and at the same time help to interpret the divergence between paralogs. In the following sections, we describe three protein families to demonstrate the diversity of evolutionary patterns encompassed in recurrent sequence evolution after independent duplication. Gene trees were constructed with IQ-TREE [53] and reconciled manually (see Material and Methods), retaining only the relevant branches with unambiguous topology (Figs. 5, 6 and 7).

#### Recurrent relocalization

Figure 5 shows the evolutionary history of Hint1/Hint2 (Histidine Triad Nucleotide Binding Proteins 1 and 2 [54]). Four independent duplications in (the ancestors of) Metazoa, Streptophyta, *Blastocystis hominis* and *Aurantiochytrium limacinum* each created paralogs with differential subcellular localization as predicted by TargetP. The Hint1 copy is almost always localized to the cytoplasm, except in *Arabidopsis thaliana*. The Hint2 copy is localized to the mitochondrion in Metazoa and *A. limacinum*, to the chloroplast in Streptophyta, and is secreted in *B. hominis*. The Hint1–Hint2 differentiation is manifested clearly in the alignment: Hint2 copies have an extended N-terminus, which corresponds to the target peptides predicted by TargetP.

It is not resolved whether the target peptide was recurrently gained or lost during evolution. Szklarczyk & Huynen (2009) have previously reported that the mitochondrial target sequence of human Hint2 was acquired in the ancestor of animals, shortly after the duplication [19]. In that case, human Hint1 maintains the ancestral subcellular localization. The argument for recurrent gain is reinforced by the highly conserved cytoplasmic localization of Hint1 copies across the four duplication events. On the other hand, the single-copy gene in *Capsaspora owczarzaki*, a species that branched off before the Metazoan duplication took place, also has a target sequence and the concomitant mitochondrial localization. This implies loss of the target peptide in Hint1 instead of gain in Hint2. The same is true for the two species that are most closely related to Streptophyta and *A. limacinum* in our tree, i.e. *Volvox carteri* and *Aplanochytrium kerguelense*. On the whole, a mix of loss and gain of target sequences might be responsible for the pattern of pervasive recurrent relocalization in Hint1/Hint2.

#### Recurrent sequence evolution reveals evolutionary constraints

In families with recurrent relocalization, a large piece of the sequence evolves recurrently (i.e. the target peptide). In Madbub, recurrent sequence evolution even involves multiple regions. Protein function can also change dramatically with few substitutions, as in the case of C4 PEPC [16]. We surveyed our output for cases of recurrent sequence evolution that are linked to specific amino acid changes.

In Sco1/Sco2 (Cytochrome C Oxidase Assembly Proteins 1 and 2 [54]), at least eight independent duplications occurred (Fig. 6). Three independent duplications had already been recognized in flowering plants, human and yeast [55]. To these we add duplications in (the ancestors of) *Branchiostoma floridae*, Mucoromycotina, *Thecamonas trahens*, *G. theta* and *Chlorella variabilis*. Several sequence features are consistently differentiated between Sco1 and Sco2 for a subset of duplications. The two most recurring features are substitutions in the mitochondrial intermembrane domain (the thioredoxin-like fold) at positions 151 and 276, being repeated in five and seven duplications, respectively. Two small indels, in the mitochondrial target peptide (10 aa) and in the mitochondrial matrix region (5 aa), also distinguish Sco1 from Sco2 in four independent duplications each.

The fact that two substitutions are repeated in five or seven out of eight duplications with similar overall divergence suggests that these positions are under the influence of natural selection. To distinguish between different types of selection, species with a single gene copy were inspected as a proxy for the ancestral state (Fig. 6). Based on this, we argue that position 276 was under purifying selection prior to the duplication as 9 out of 10 single-copy genes carry an asparagine. Purifying selection has maintained the asparagine in almost all Sco1 copies. For Sco2, repeated replacement of the asparagine by various other amino acids suggests a relaxation of purifying selection with respect to the ancestral state. Apparently, Sco2 copies no longer require an asparagine for their function. At position 151, the explanation is the other way around. There is no single residue that represents the ancestral state, indicating a low level or absence of purifying selection. After five independent gene duplications, Sco1 abruptly conserves a lysine, whereas Sco2 still carries one of various amino acids. This reveals recurrent positive selection for a lysine at position 151 in the Sco1 copy.

The precise functional differentiation between Sco1 and Sco2 in different species remains unknown despite various direct experimental studies [56–58]. New evidence suggests that they may have “distinct antioxidant capacity” in addition to their partially redundant roles in cytochrome c oxidase assembly [59]. Both are localized to the mitochondrion, but their specific location within the mitochondrial matrix could be different as a consequence of the indels in the target sequence and matrix region. Our evolutionary analysis has also identified two residues which have not been considered yet and which could help disentangle their different biological roles.

#### Possible recurrent complexification of a molecular machine

In 2012, the Thornton lab published remarkable results on a molecular machine, the vacuolar (V-) ATPase, which has increased in complexity through the duplication-degeneration-complementation mechanism [34]. In fungi, a duplication of vma3 encoding the c subunit of the proteolipid ring created vma11 encoding a specialized c’ subunit. Concurrently, the composition of the full membrane ring changed from c_7_c”_1_ to c_6_c’_1_c”_1_, which was discovered only recently using cryo-EM [60, 61]. The third subunit, c” encoded by vma16—which serves as an anchor point between the rotating ring and the rest of the ATPase—is related to vma11/vma3 by a duplication prior to LECA. Finnigan et al. (2012) showed that the ancestral vma11/vma3 protein can take in the positions of both vma11 and vma3. However, through the neutral loss of complementary interaction interfaces in vma11 and vma3, the two subunits have become indispensable. The loss of each interface was recapitulated by two point mutations in the first transmembrane *α*-helix (TM1) that are still conserved in extant fungi: V15F in vma11 and M22I in vma3.

In our dataset, at least eight independent duplications were found to mimic the vma11–vma3 differentiation in fungi (Fig. 7). While these duplications initially appeared to be very recent, with an extended genome set we find that only the duplication in *B. hominis* is potentially species-specific (Suppl. Figure S6). This is in line with the degree of differentiation observed between the sister paralogs. Independent duplications occurred in (the ancestors of) Platyhelminthes (*Schistosoma mansoni*), Chromadorea (*C. elegans* and *Brugia malayi*), *B. hominis*, Phaeophyceae (*Ectocarpus siliculosus*), Cryptosporidia (*Cryptosporidium parvum Iowa*), Spirotrichea (*Oxytricha trifallax*), Oligohymenophorea (*Tetrahymena thermophila*) and Diplomonadida (*Giardia intestinalis*).

The pattern of recurrent sequence evolution is more distributed over the entire sequence compared with the two previous examples, but it contains valuable information. The seven positions that are most consistent with the fate differentiation all lie on the sides where interaction with adjacent subunits in clockwise or anti-clockwise direction takes place, i.e. TM1–TM2 or TM3–TM4, respectively (Fig. 7). In contrast, the vacuolar sections show little recurrence despite the fact that they contain a comparable number of variable positions. The enrichment of recurrent substitutions around the interaction interfaces suggests their degeneration resulted in complexification of the V-ATPase in eight eukaryotic groups in a similar manner as it did in fungi.

In several lineages, more than one duplication took place, suggesting additional specialization of the proteolipid ring subunits (Suppl. Figure S6). In the diplomonad *Spironucleus salmonicida*, the vma3-like copy underwent a second round of duplication followed by substantial sequence divergence. In Kinetoplastida, six species have retained three gene copies resulting from two duplications, one before the split from *Perkinsela spp.* and one afterwards. We do not know what fates these three copies represent, so it would be interesting to study the composition of the V-ATPase membrane ring in one of these species. The proteolipid ring subcomplex apparently leaves ample room for complexification.

## Conclusion

We have developed new methodology to detect recurrent sequence evolution between independently arisen paralogs. The entire framework is modular, which makes it amenable to changes in particular steps. For instance, the identification of duplications in a family could be performed by phylogenetic reconstruction instead of the network approach, as we have manually done for Hint1/Hint2, Sco1/Sco2 and vma11/vma3. In the future, the framework could also be extended to incorporate weighing of recurrent patterns according to site heterogeneity or amino acid substitution rates.

Gene conversion where one paralogous copy replaces another could create a peculiar pattern of “independent” gene duplication. In particular loci that are ancestrally duplicated would appear as lineage-specific duplications. However, given that gene conversion maintains sequence identity between paralogs (e.g. in order to maintain cytoplasmic ribosomal protein in yeast), we do not expect this category of pseudo-independent duplicates to display recurrent *divergent* sequence changes.

In contrast to orthologs [6, 7], paralogs frequently undergo functional differentiation that results in recurrent sequence evolution. Nearly neutral evolution might still explain a substantial fraction of recurrent patterns, but for families with more than two independent duplications with consistent recurrent changes, this explanation becomes increasingly unlikely. One reason to surmise functional differentiation is that these families with pervasive recurrent sequence evolution include known proteins with different functions (Table 1, Suppl. Data). Another reason is that a particular type of recurrent functional differentiation, i.e. relocalization, was observed on a large scale. This type is picked up by our framework because a target peptide is identified as the recurring sequence element (as in Hint1/Hint2). Not all patterns of recurrent sequence evolution can be linked to function as easily, but they might inspire biochemical studies aimed at extracting such function. We found striking signatures of selection at the residue level (e.g. positive selection on 151K in Sco1/Sco2) and at the domain level (e.g. relaxed purifying selection on interaction interfaces in vma11/vma3). All these patterns underscore the evolutionary significance of gene duplication in the expansion of the eukaryotic functional repertoire.

While the genome sequencing revolution continues unabated, functional annotation of predicted proteins remains largely dependent on tools for detecting sequence homology. For one-to-one orthologs in different species, the orthology conjecture has warranted functional equation. In the present study we have explored the functional relationship between independently derived pairs of paralogs existing in different species. We have found families with extensive recurrent sequence evolution, where duplicates are likely to have independently acquired one of two specific fates. In those cases, it would be legitimate to transfer the functional annotations from each member of a functionally characterized duplicate pair to each of the independent duplicates in the other lineage with the predicted equivalent fate. Such two-to-two functional projection is a more precise extension of the classical one-to-one projection between orthologs and could assist in the annotation of many paralogs.

So what explains the pervasiveness of recurrent sequence evolution in particular eukaryotic protein families? Gene duplication is a common mutation [62] so mutation rate heterogeneity does not suffice as answer. We think that some genes such as vma11/vma3 and Madbub, are poised for recurrent sequence evolution because they provide cellular functions that can easily be subdivided or recruited for slightly different purposes, thus ensuring evolutionary preservation of duplicates (cf. [63]). The exact role of neo- and subfunctionalization in retaining independently duplicated genes remains an open problem. While we find that most paralogs diverge asymmetrically, this does not necessarily relate to an asymmetry at the level of protein function. Perhaps neo- or subfunctionalization can be further characterized through gene expression analysis (see e.g. [64]), which is the context where these terms were coined [9, 14, 65]

## Methods

### Definition of gene families

Our dataset was constructed from the protein sequences of 1,450,432 genes from 90 diverse eukaryotic species, as detailed in Van Hooff et al. (2017; Appendix Table S2 [66]). To obtain a workable set of eukaryotic gene families, genes were assigned to PANTHER v9.0 families and subfamilies [29]. To this end, each gene was compared to the hidden markov model of every PANTHER family and subfamily using ‘hmmscan’ from the HMMer package [67]. 1,015,711 genes (70%) were successfully assigned to the best-matching PANTHER family using the default HMMer bit-score cutoff of 22. This way, 7068/7180 (98.4%) families and 45,038/52,768 (85.4%) subfamilies were covered. From the 51,206 (sub)families (or simply “families”), those with more than 10 duplicate pairs were selected for analysis (3388 or 6.6%). Our framework could successfully analyze 2883 of the selected families (85.1%).

A multiple sequence alignment was constructed for each family using MAFFT v.7.271 with the ‘E-INS-i’ option that gives high accuracy while making the fewest assumptions [68]. Single-copy genes or genes with more than two copies are included in this step, because they could aid aligning in cases where duplicate pairs are highly divergent and would otherwise be difficult to reconcile [23]. For the inference of duplications, alignments were trimmed using trimAl v1.2 with the ‘gappyout’ option that automatically sets the threshold for trimming based on gaps and similarities distribution [69]. For the identification of fates, the untrimmed alignments were used to include potential recurrent patterns outside conserved regions. Nonetheless, using trimmed alignments to predict fates of paralogs did not alter our overall results (Suppl. Figure S7).

### A framework for identifying recurrent sequence evolution after independent duplication

The framework consists of two distinct modules that run separately (Fig. 1; see “Software” in the Supplementary Material for programmes and programme languages used). On one side, independent duplication events in a gene family are inferred. Multiple paralog pairs may originate from a single duplication event in the common ancestor, or from several independent duplication events. On the other side, the fates of all paralogs are determined. Divergence between sister paralogs results in two distinct fates for each pair. Multiple paralog pairs can have the same two fates or a multiple of two; in the limit, each gene represents a unique fate.

The first step is the analysis of substitution patterns in aligned quartets of sequences (two pairs of duplicates), building on existing methodology [7, 70] (“Substitution analysis in aligned quartets”). In the second step, information from each quartet is integrated for the entire family through networks, one for duplications and one for fates (“Integration of quartets in networks”). Clustering these networks eventually yields two measures that describe the pervasiveness (*P*) and the magnitude ($\overline {Z}_{F}$) of recurrent sequence evolution in a family (“Scoring recurrent sequence evolution” sections).

### Substitution analysis in aligned quartets

For any four homologous genes, there are three possible unrooted trees corresponding to different evolutionary relationships (*Q*, *R* and *S*; Fig. 1). The tentative phylogeny is the scenario that is best supported by the alignment. Under Maximum Likelihood, support for a single duplication as origin of two paralog pairs in a quartet, *D* (i.e. NOT *Q*), can be defined by the likelihood of the particular trees (i.e. 1−*w*_*Q*_). The expected likelihood weight (ELW; [71]) of each tree was calculated using TREE-PUZZLE [72] with the LG +*Γ*10 model [73]. Using this model instead of the most optimal substitution model per family did not bias our results (Suppl. Figure S8).

The evolutionary scenarios *Q*, *R* and *S* are also used to define fate similarity. Here, a difference in the number of sites that support *R* or *S*—rather than expected likelihood weights as for the phylogenetic signal—suggests that each paralog is more similar to one specific paralog of the other species. If the difference is substantial it could imply two-to-two functional correspondence between paralogs from different species. Such fate similarity $\left (F=\frac {|n_{R}-n_{S}|}{n_{Q}+n_{R}+n_{S}}\right)$ is trivial in the case that paralog pairs are derived from the same duplication event (*R* or *S* being the primary phylogenetic signal). However, if the paralog pairs are derived from independent duplication events (*Q* being the primary phylogenetic signal) such fate similarity represents *recurrent sequence evolution*. This notion of recurrent sequence evolution is different from previous studies on orthologs [7] because not the total number of recurrent substitutions (*n*_*R*_+*n*_*S*_), but rather a bias therein is measured (a “secondary phylogenetic signal” |*n*_*R*_−*n*_*S*_|).

### Integration of quartets in networks

Detecting recurrent sequence evolution between three or more species adds a layer of complexity. We do not want to simply count the number of quartets with significant recurrent sequence evolution because ancient duplications give rise to many paralog pairs in a family. Recurrent sequence evolution happens between independent duplications, so all species that share the same ancestral duplication should behave uniformly. If the human paralogs in Fig. 1 acquired the same fate as the yeast paralogs, then the frog paralogs should also have acquired the same fate as yeast paralogs, since human and frog paralogs have the same fates by virtue of the ancestral duplication. This property of transitivity can be exploited in networks.

First, to find duplications, a network is constructed with species as nodes. The edges between species are given by *D*, which results in a complete graph. Clusters are obtained with the cluster editing algorithm PEACE [74], revealing which species share an ancestral duplication (threshold *θ*=0.6, see “Validation for Network Clustering” in Supplementary Material).

To find all fates in a family, a second network is constructed with genes as nodes. Edges are drawn between paralogs from different species that have the same fate. Thus, two edges are drawn for each quartet (e.g. from Human_a to Yeast_c and from Human_b to Yeast_d in Fig. 1); both are weighted by fate similarity *F*. Clusters are obtained with the Markov Clustering algorithm (inflation = 10, expansion = 5 [75]), revealing which genes have similar fates. Genes deriving from the same duplication event almost always cluster together, whereas clusters that comprise multiple complete independent duplications signifying recurrent sequence evolution are far less common.

### Scoring recurrent sequence evolution

To improve the fate clustering, the alignment of each family was bootstrapped 100 times, sampling with replacement and redoing the quartet analysis and fate clustering every iteration. The most robust, non-overlapping clusters of fates are determined using the jaccard coefficient [76]. In this manner, bootstrapping potentially filtered out clusters that depend on a few specific positions in the alignment (fate clusters did not change substantially; Suppl. Figure S9). Here we arrive at a final hypothesis for the duplication events and fates present in the family (bottom center in Fig. 1).

We define the pervasiveness of recurrent sequence evolution in a family *P* as the number of duplications for which all genes end up in the same two fates. For this prevailing differentiation, we also calculate the magnitude of recurrent sequence evolution by taking the average of *Z*_*F*_ of all quartets consisting of two independently duplicated pairs (i.e. those with recurrent sequence evolution). Here, *Z*_*F*_ is the standard score for *F*, or rather of the absolute difference *dF*=|*n*_*R*_−*n*_*S*_|. Assuming that patterns follow a Bernoulli distribution along an alignment with length *l*, the variance of *dF* can be calculated and then *Z*_*F*_, as described in [70]:

1 $$ {} V(\mathit{dF})=\sum\limits_{x\in R,S}x\left(1-\frac{x}{l}\right)+\sum\limits_{x,y\in R,S|y\ne x}\frac{2xy}{l}  $$

2 $$ Z_{F}=\frac{{dF}}{\sqrt{(V({dF})}}  $$

### Quartets of whole genome duplications

For a small subset of species in our dataset, it has been previously assessed and catalogued whether paralogs derive from a particular whole genome duplication. For *S. cerevisiae*, the Yeast Gene Order Browser [77] (YGOB) is used to identify paralogs originating from the yeast WGD. The OHNOLOGS database [78] is used to retrieve *Homo sapiens*, *Mus musculus* and *D. rerio* ohnologs from the vertebrate WGD (2R) and again *Danio rerio* from the bony fish WGD (3R). To complete the quartets, other post-WGD species are added: *Candida glabrata* is included in the yeast WGD, *X. tropicalis* in 2R, and *Takifugu rubripes* in 3R. Single duplication quartets from all families containing these particular species can then be subdivided into “WGD” quartets and “Other” quartets (Figs. 3–4). For instance, there are 310 single duplication quartets in the entire set of families that consist of *S. cerevisiae* and *C. glabrata* paralogs. Forty-six of these quartets were classified as WGD quartets based on the annotation of the *S. cerevisiae* pair in YGOB. In total, 616 WGD quartets and 2322 Other quartets were identified; due to additional criteria specific to the analyses of asymmetry and subcellular localization, some of these were not used (see Figs. 3–4).

### Identifying recurrent patterns in the alignment

Up to this point our framework has been agnostic with respect to which amino acid changes contribute to recurrent sequence evolution, as only the *D* and *F* scores of quartets are integrated in the networks. Now we can go back to the alignment and identify the positions that are consistently differentiated between paralogs from independent duplications (Suppl. Figure S2). This is not straight-forward owing to the complex and diverse evolutionary histories of families (Suppl. Figure S1).

For every pair of duplicates, the frequency of informative patterns at each position in the alignment is computed. Positions that support the overall fate prediction more often than that they contradict it, are given warm colors (yellow, red or pink; Suppl. Figure S2). Positions that contradict the overall fate prediction more often are given cold colors (blue, cyan or green). The specific colors (i.e. yellow versus pink or blue versus green) are used to indicate whether positions display a particular asymmetry. From the colored alignment, a sequence logo can be made, summarizing the relative frequency of recurrent patterns at each position in the alignment for all paralog pairs (Suppl. Figure S2).

The above approach is complementary to some other methods used to determine interesting residue differences between paralogs [40–42]. In these methods, differential conservation between two post-duplication branches is what reveals the interesting residues, rather than recurrence between multiple independent duplications.

### Manual gene family analysis

For Hint1/Hint2, Sco1/Sco2 and vma11/vma3, the trimmed alignments were manually curated using JalView [79], removing highly diverged sequences and sequences that were (almost) identical to another sequence. Gene trees were constructed with IQ-TREE v1.5.5 [80] using ModelFinder [53] and ultrafast bootstrap approximation [81] (see Suppl. Figures S5-6). The trees were rooted between Unikonta and Bikonta as far as possible. The gene trees were then reconciled with the species tree leading to a manual prediction of independent duplication events. In Figs. 5, 6 and 7, only duplication nodes that could be coherently projected onto the species tree were kept during the analysis. Duplication nodes are annotated between two adjacent branches in the gene tree consisting of the same species. Gene trees are inherently noisy predictions, so small deviations from the ideal pattern are still acceptable. For the purpose of reproducibility and transparency, the gene trees of Sco and vma prior to reconciliation are provided in the Supplementary Material (Suppl. Figures S5-6).

For vma11/vma3, we also used an extended dataset with genes from 210 eukaryotic species in total. First, we searched for genes that matched the HMM model of vma11/vma3 (PTHR10263) using ‘hmmscan’ [67]. After a quick alignment performed by ClustalW v2.1 [82], the orthologous group containing vma11/vma3 was obtained by inspection of the tree in FigTree v1.3.1 [83]. Then, the alignment and gene tree were reconstructed as described above.

## Supplementary information

**Additional file 1** Supplementary Material (pdf format), contains Supplementary Methods and Supplementary Figures. Supplementary Methods: section describing validation of network clustering and short overview section of used programmes and programming languages. Supplementary Figures 1-9: additional data to support the manuscript (see text for references).

## Data Availability

The datasets generated and analysed during the current study are available in a public repository based at Theoretical Biology and Bioinformatics, Utrecht University: https://bioinformatics.bio.uu.nl/snel/support/Supplementary_Data.zip. The source code for the main framework is available from the same location: https://bioinformatics.bio.uu.nl/snel/support/RecurrentEvolution.zip.

## Abbreviations

PEPC: Phosphoenolpyruvate carboxylase
ML: Maximum likelihood
RSE: Recurrent sequence evolution
WGD: Whole genome duplication
LECA: Last eukaryotic common ancestor
aa: Amino acid
ELW: Expected likelihood weight
YGOB: Yeast gene order browser
