# Supplementary material for: Recurrent sequence evolution after independent gene duplication
Source: BMC Evol Biol. 2020 Aug 8;20:98. doi: 10.1186/s12862-020-01660-1 (PMC7414715; doi:10.1186/s12862-020-01660-1)
Supplement: Supplementary file 1 — Additional file 1 Supplementary Material (pdf format), contains Supplementary Methods and Supplementary Figures. Supplementary Methods: section describing validation of network clustering and short overview section of used programmes and programming languages. Supplementary Figures 1-9: additional data to support the manuscript (see text for references). [file 12862_2020_1660_MOESM1_ESM.pdf]

Supplementary Material to Recurrent Sequence Evolution After Independent Gene Duplication

1 Supplementary Methods

1.1 Validation for Network Clustering

In the current study we predicted independent duplications from a network (see "Integration of Quartets in Networks" in main paper). This approach was validated through manual tree reconstruction for six gene families which were chosen because they had been previously proposed as examples of recurrent subfunctionalization (Madbub and AP1-β/AP2-β, i.e. Dacks et al. 2008, Suijkerbuijk et al. 2012) or because their copy number distribution hinted at multiple independent duplications (Cox11/Mettl17, Bet1/Bet1l, Shmt1/Shmt2 and Tmem50a/Tmem50b). Cox11/Mettl17 actually revealed a single pre-LECA duplication in the gene tree, thus serving as a control.

From the six gene trees, golden standard sets were manually constructed of paralog clusters that unambiguously derived from the same duplication events. The $F_{k \to 2}$ statistic from Drew et al. (2017) was used to assess the congruence of clusters obtained from networks with these golden standard sets. We did not use $F_{grand,\ k\text{-}clique}$ (Drew et al. 2017), because it penalizes too heavily in cases where a large cluster is complete except for one species. Seven clustering methods were tested (code written in R using igraph package; Csardi and Nepusz 2006): a simple edge threshold, Markov Clustering algorithm (MCL package; Jäger 2015), HCS (Highly Connected Subgraphs), label propagation, community clustering with fastgreedy and walktrap approaches, and PEACE (Böcker et al. 2008). For each of these, 101 different edge thresholds in the range $[0.0, 1.0]$ were tested.

The best performance was obtained with PEACE using a threshold at $\theta = 0.6$. When $D \geq 0.667$, n_Q is not the greatest of n_Q, n_R and n_S (quartet represents single duplication), while $D \leq 0.5$ gives that Q is definitely in the majority of informative positions (quartet represents independent duplications). Using the six families mentioned above, we confirmed that this threshold leads to very similar duplications being identified as with manual gene tree reconciliation (average $F_{k \to 2} = 0.88$). In fact, the precise threshold is not so important because we use Maximum Likelihood weights rather than counts (w_X versus n_X for $x \in Q, R, S$), which produces even more binary values of D ($D \to 0$ or $D \to 1$).

For the clustering of fates from a network, the above approach could not be employed since we only had information of fates for Madbub. MCL was chosen (inflation = 10, expansion = 5, no threshold), because it distinguished most Mad and Bub fates in Madbub ($F_{k \to 2} = 0.93$) and because it is a very commonly used clustering algorithm.

1.2 Software

All scripts were written in bash, python, snakemake (Köster and Rahmann 2012) or R with packages igraph (Csardi and Nepusz 2006) and MCL (Jäger 2015). FigTree v1.3.1 (Rambaut 2009), JalView v2 (Waterhouse et al. 2009) and Cytoscape v3.5.1 (Shannon et al. 2003) were used to look at trees, alignments and networks, respectively. Aligning was performed with MAFFT v7.271 E-INS-i (Katoh and Standley 2013) or ClustalW v2.1 (Thompson et al. 2003), trimming with trimAl v1.2 (Capella-Gutiérrez et al. 2009), duplication detection in quartets with TREE-PUZZLE (Schmidt et al. 2002) and tree reconstruction with IQ-TREE v1.5.5 (Nguyen et al. 2014). For PEACE, the C++ code provided by Böcker et al. (2008) was used. For $F_{k\to 2}$, the python code from (Drew et al. 2017) was used. Figures were made with Inkscape (Harrington 2005) and TreeGraph v2.14 (Stöver and Müller 2010).

2 Supplementary Figures

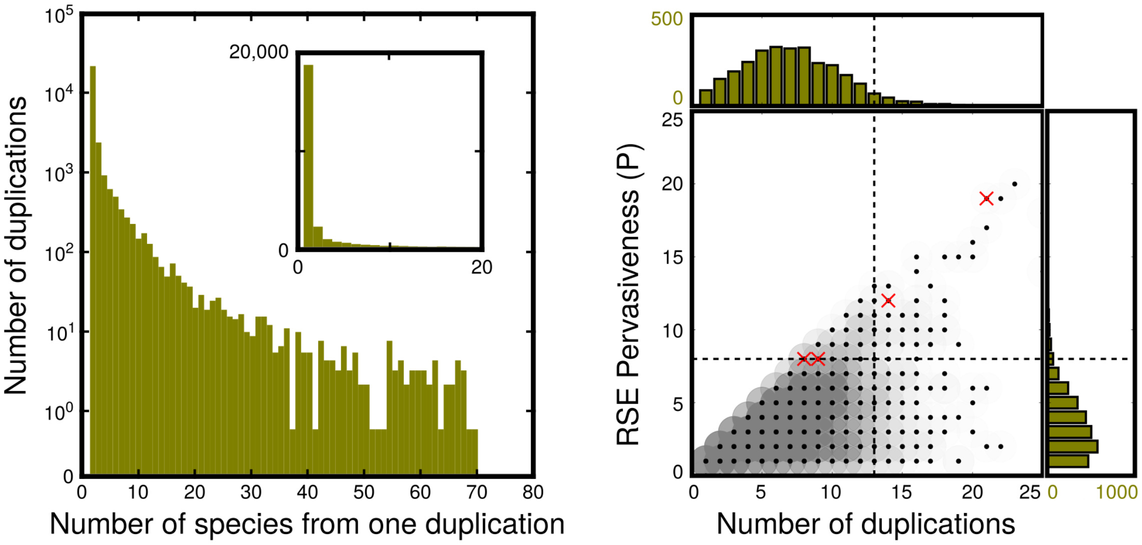

Figure 1: (a) Size (number of species) of all duplications in the dataset as a proxy for their age and (b) total number of duplications versus number of recurrently evolving duplications (P) per family. Most duplications that we consider in our dataset (i.e. which are still retained in two copies) are taxon-specific. There is substantial variation in the number of duplications per family.

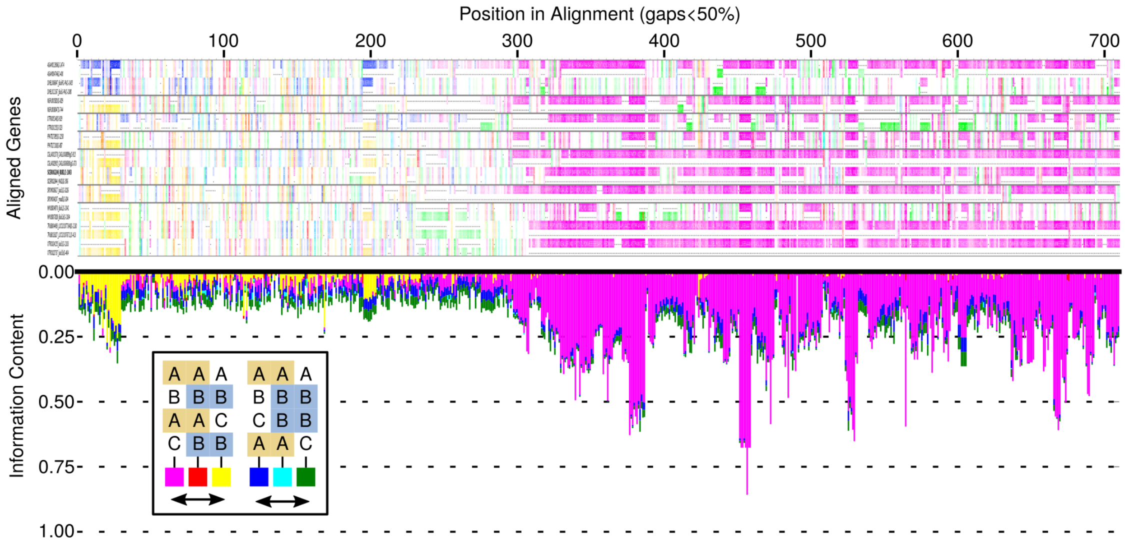

Figure 2: Identification of positions most consistent with recurrent fate prediction in Madbub. On the top, a compressed alignment is shown for the duplicates involved in recurrent sequence evolution. On the bottom, the summarizing sequence logo is shown. Colors denote the most frequently observed informative pattern (alignment) and relative frequency of each pattern (sequence logo). Fates are ordered ABAB, so patterns in R here represent the consistent signal while patterns in S represent the inconsistent signal. The N-terminal KEN box lights up in yellow (ABAC), the kinase domain in pink (ABCB).

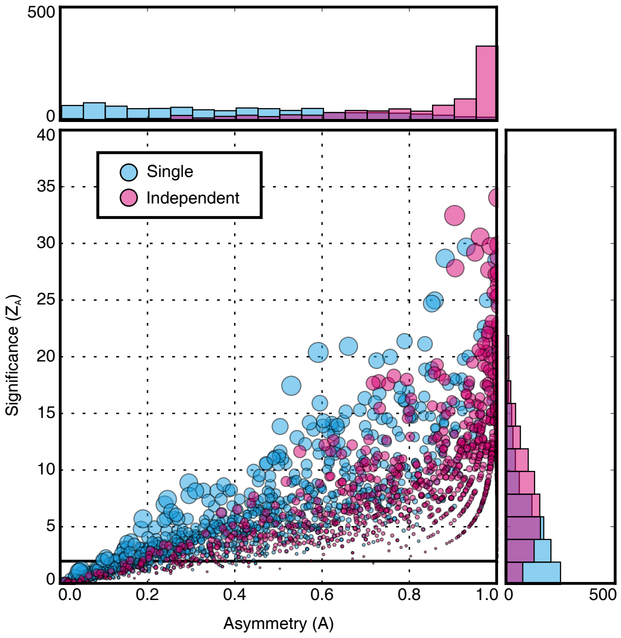

Figure 3: Asymmetry in paralog differentiation ($A = |t - u|$) versus significance of asymmetry (Z_A) for single and independent duplication quartets (see Figure 3 in main paper). Disks are scaled by the number of positions that support the fate differentiation (i.e. n_R if R is the dominant fate differentiation). Z_A is calculated in the same way as Z_F (see Material and Methods in main paper):
$V(A) = t\left(1 - \frac{t}{l}\right) + u\left(1 - \frac{u}{l}\right) + \frac{2tu}{l}$, and then $Z_A = \frac{A}{\sqrt{V(A)}}$.

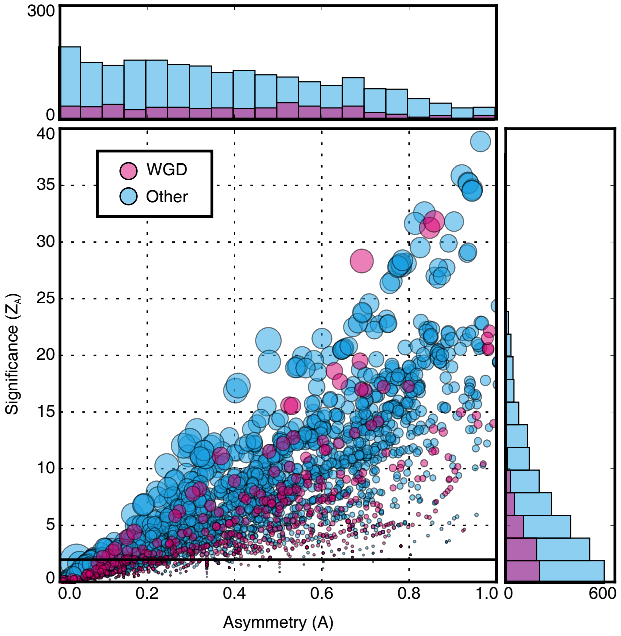

Figure 4: Asymmetry in paralog differentiation between single duplication quartets consisting of ohnologs ("WGD") and non-ohnologs ("Other"). In contrast to Figure 3, WGD quartets do not seem to have a different distribution of asymmetry than Other quartets. WGDs contain more recently duplicated genes (smaller disks) and therefore fewer WGD quartets obtain significant asymmetry than Other quartets.

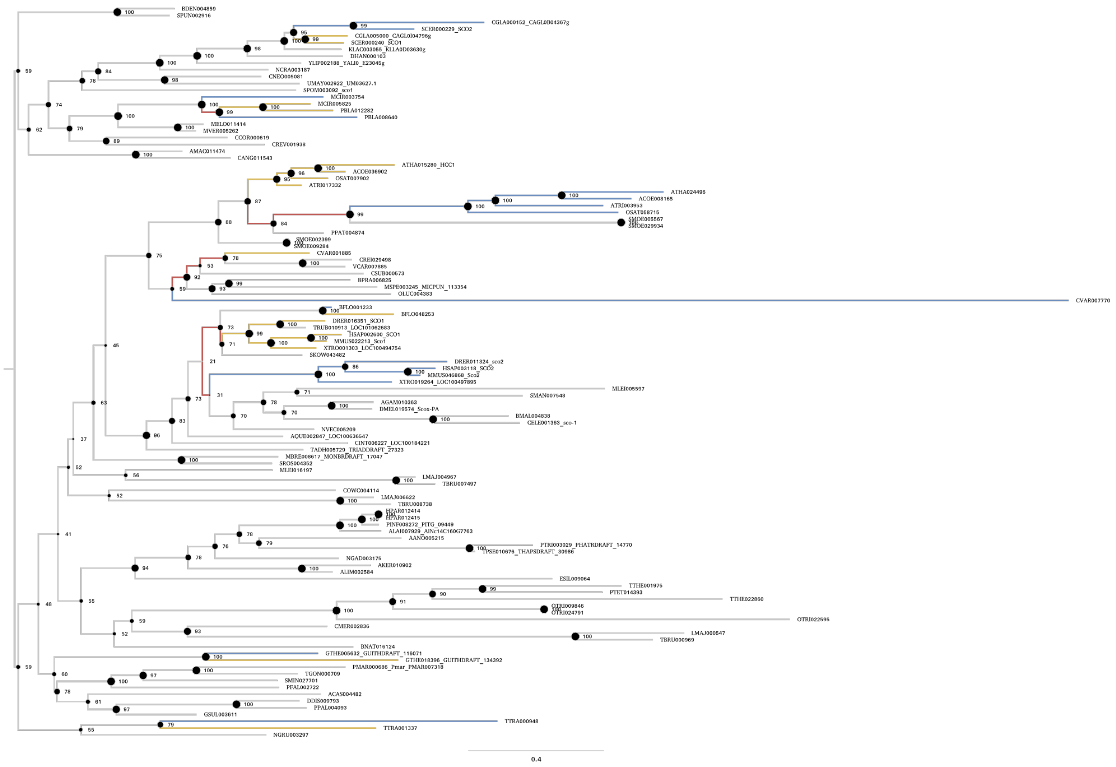

Figure 5: Gene tree of Sco1/Sco2. Sequences were aligned with MAFFT (Katoh and Standley 2013) and trimmed with trimAl (Capella-Gutiérrez et al. 2009); tree was constructed with IQ-TREE using ModelFinder (Kalyaanamoorthy et al. 2017) and rooted between fungi and all other eukaryotes. If multiple genes were almost identical, only one was kept. Genes that were highly dissimilar to all other genes were also removed. Branches corresponding to Sco1 or Sco2 are colored yellow or blue, respectively (following the general color scheme for fates).

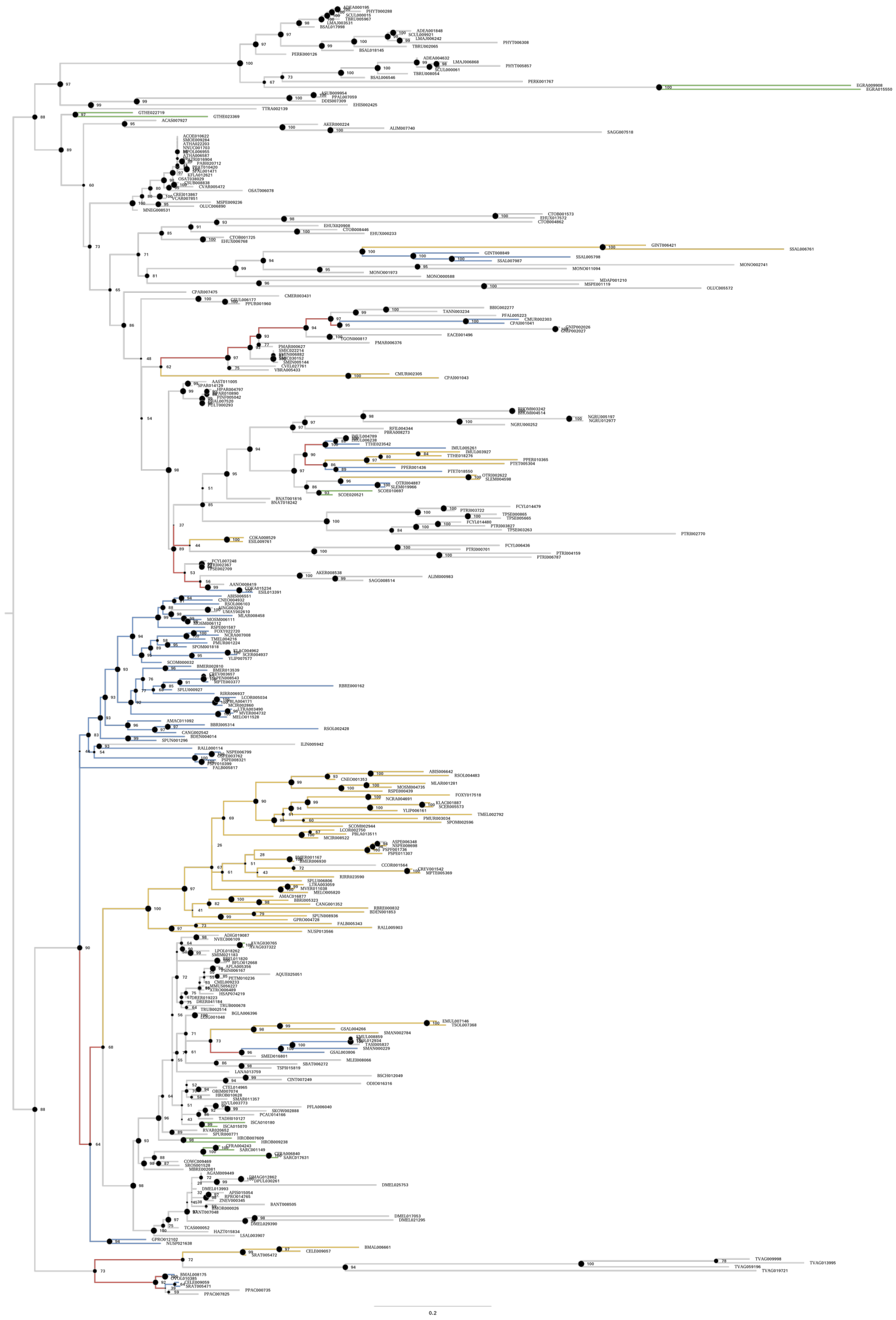

Figure 6: Gene tree of vma11/vma3. Sequences were aligned with MAFFT (Katoh and Standley 2013) and trimmed with trimAl (Capella-Gutiérrez et al. 2009); tree was constructed with IQ-TREE using ModelFinder (Kalyaanamoorthy et al. 2017) and rooted between Unikonta and Bikonta (only TVAG or *Trichomonas vaginalis* genes appear on the wrong side of the root). If multiple genes were almost identical, only one was kept. Genes that were highly dissimilar to all other genes were also removed. Branches corresponding to vma11 or vma3 are colored yellow or blue, respectively (following the general color scheme for fates). Duplicates that are only present in the extended genome set are colored green to indicate that these may also represent sequences with the vma11 or vma3 fate. The two duplications in Kinetoplastida (see main text) are at the top.

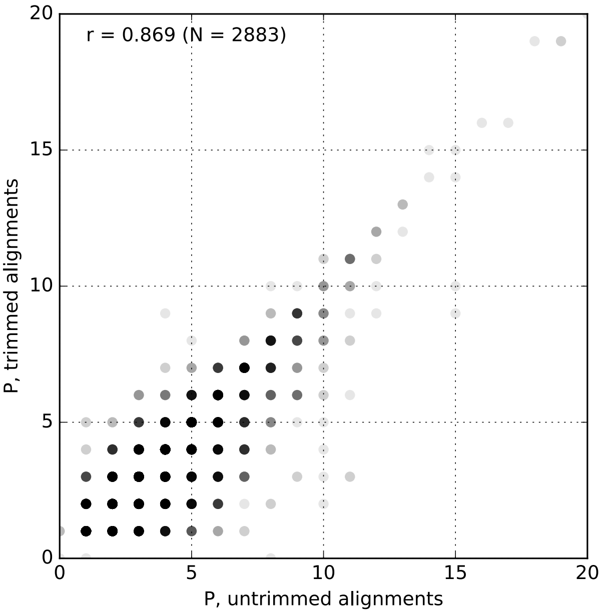

Figure 7: Trimming alignments for fate prediction does not impact the prevalence of recurrent sequence evolution. Fate clusters were now constructed after alignments were trimmed (i.e. from the same trimmed alignments used to predict duplications). These fate clusters were then used along with the original duplication clusters to obtain a second P score for each family. Pearson correlation coefficient $r = 0.869$.

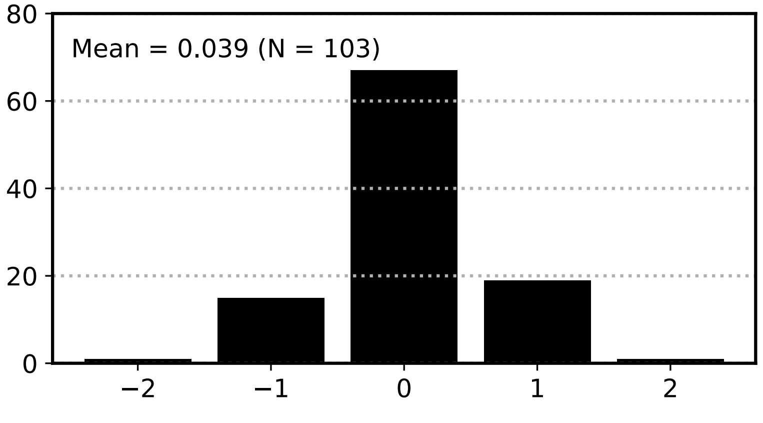

Figure 8: The LG+Γ10 model does not result in a systematic bias in the assessment of recurrent sequence evolution compared to the optimal model for a protein family. For a random subset of 103 families, the optimal model was predicted (ModelFinder "-MF" and "-TESTONLY"; Kalyaanamoorthy et al. 2017) from the family multiple sequence alignment after trimming (i.e. including all genes). Only FreeRate ("+R") models were dismissed as they could not be implemented in TREE-PUZZLE. The optimal sequence models were LG (97), VT (4), Blosum62 (1) and WAG (1). In addition to 4 gamma distributed rate categories (+G4) with optimized alpha, 29 families required a mixed rate model (+I+G4) where a proportion of invariant sites is assumed. Finally, for 36 families, the background amino acid distribution was estimated from the alignment (+F) instead of using the standard distribution of the specific substitution model.

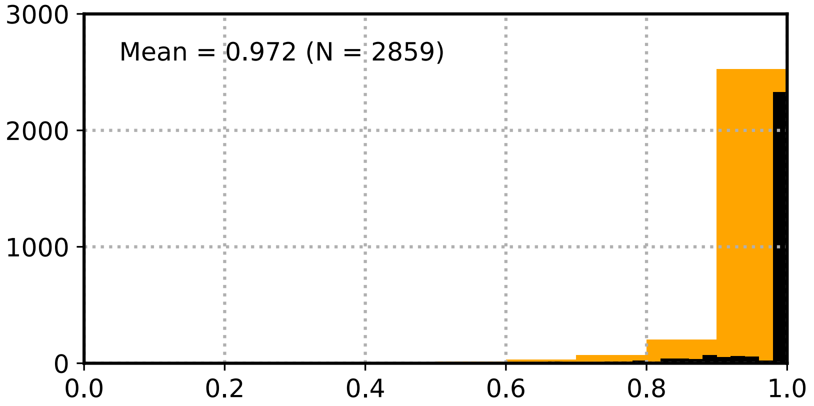

Figure 9: Effect of bootstrapping on fate clusters. To obtain more support for the fate clustering, the clustering was also performed for 100 bootstrapped alignments per family (see Material and Methods in main manuscript). The fate clusters derived from the original alignments were compared with those based on the bootstrap ensemble (i.e. those used by the framework described in the main manuscript). The $F_{k\to2}$ score was again used to quantify the similarity between clusters. For a few families (24 of 2883), $F_{k\to2}$ is undefined because all clusters in at least one of the two sets are size 1 (one fate per gene). The distribution of $F_{k\to2}$ values is shown at two different bin widths (orange, bw = 0.1; black, bw = 0.02).

References

Böcker, S., Briesemeister, S., and Klau, G. W. (2008). Exact Algorithms for Cluster Editing: Evaluation and Experiments. In McGeoch, C. C., editor, *Experimental Algorithms*, pages 289–302, Berlin, Heidelberg. Springer Berlin Heidelberg.

Capella-Gutiérrez, S., Silla-Martínez, J. M., and Gabaldón, T. (2009). trimal: a tool for automated alignment trimming in large-scale phylogenetic analyses. *Bioinformatics*, 25(15):1972–1973.

Csardi, G. and Nepusz, T. (2006). The igraph software package for complex network research. *InterJournal*, Complex Systems:1695.

Dacks, J. B., Poon, P. P., and Field, M. C. (2008). Phylogeny of endocytic components yields insight into the process of nonendosymbiotic organelle evolution. *Proceedings of the National Academy of Sciences*, 105(2):588–593.

Drew, K., Lee, C., Huizar, R. L., Tu, F., Borgeson, B., McWhite, C. D., Ma, Y., Wallingford, J. B., and Marcotte, E. M. (2017). Integration of over 9,000 mass spectrometry experiments builds a global map of human protein complexes. *Molecular Systems Biology*, 13(6).

Harrington, B. (2004-2005). Inkscape.

Jäger, M. L. (2015). Mcl package.

Kalyaanamoorthy, S., Minh, B. Q., Wong, T. K. F., von Haeseler, A., and Jermiin, L. S. (2017). Modelfinder: fast model selection for accurate phylogenetic estimates. *Nature Methods*, 14:587–.

Katoh, K. and Standley, D. M. (2013). MAFFT Multiple Sequence Alignment Software Version 7: Improvements in Performance and Usability. *Molecular Biology and Evolution*, 30(4):772–780.

Köster, J. and Rahmann, S. (2012). Snakemake—a scalable bioinformatics workflow engine. *Bioinformatics*, 28(19):2520–2522.

Nguyen, L.-T., Schmidt, H. A., von Haeseler, A., and Minh, B. Q. (2014). IQ-TREE: a fast and effective stochastic algorithm for estimating maximum-likelihood phylogenies. *Molecular Biology and Evolution*, 32(1):268–274.

Rambaut, A. (2009). FigTree v1.3.1. *http://tree.bio.ed.ac.uk/software/figtree/*.

Schmidt, H. A., Strimmer, K., Vingron, M., and von Haeseler, A. (2002). Tree-puzzle: maximum likelihood phylogenetic analysis using quartets and parallel computing. *Bioinformatics*, 18(3):502–504.

Shannon, P., Markiel, A., Ozier, O., Baliga, N. S., Wang, J. T., Ramage, D., Amin, N., Schwikowski, B., and Ideker, T. (2003). Cytoscape: A Software Environment for Integrated Models of Biomolecular Interaction Networks. *Genome Research*, 13(11):2498–2504.

Stöver, B. C. and Müller, K. F. (2010). TreeGraph 2: combining and visualizing evidence from different phylogenetic analyses. *BMC bioinformatics*, 11(1):7.

Suijkerbuijk, S. J., van Dam, T. J., Karagöz, G. E., von Castelmur, E., Hubner, N. C., Duarte, A. M., Vleugel, M., Perrakis, A., Rüdiger, S. G., Snel, B., and Kops, G. J. (2012). The Vertebrate Mitotic Checkpoint Protein BUBR1 Is an Unusual Pseudokinase. *Developmental Cell*, 22(6):1321–1329.

Thompson, J. D., Gibson, T. J., and Higgins, D. G. (2003). Multiple sequence alignment using ClustalW and ClustalX. *Current Protocols in Bioinformatics*, (1):2–3.

Waterhouse, A. M., Procter, J. B., Martin, D. M. A., Clamp, M., and Barton, G. J. (2009). Jalview Version 2—a multiple sequence alignment editor and analysis workbench. *Bioinformatics*, 25(9):1189–1191.
